# Risk Signature of Cancer-Associated Fibroblast–Secreted Cytokines Associates With Clinical Outcomes of Breast Cancer

**DOI:** 10.3389/fonc.2021.628677

**Published:** 2021-07-28

**Authors:** Chunxiao Sun, Siwei Wang, Yuchen Zhang, Fan Yang, Tianyu Zeng, Fanchen Meng, Mengzhu Yang, Yiqi Yang, Yijia Hua, Ziyi Fu, Jun Li, Xiang Huang, Hao Wu, Yongmei Yin, Wei Li

**Affiliations:** ^1^Department of Oncology, The First Affiliated Hospital of Nanjing Medical University, Nanjing, China; ^2^Department of Thoracic Surgery, Jiangsu Key Laboratory of Molecular and Translational Cancer Research, Nanjing Medical University Affiliated Cancer Hospital & Jiangsu Cancer Hospital & Jiangsu Institute of Cancer Research, Nanjing, China; ^3^Jiangsu Key Lab of Cancer Biomarkers, Prevention and Treatment, Collaborative Innovation Center for Personalized Cancer Medicine, Nanjing Medical University, Nanjing, China; ^4^Department of Oncology, Sir Run Run Hospital of Nanjing Medical University, Nanjing, China

**Keywords:** breast cancer, prognostic signature, cancer-associated fibroblast, cytokine, tumor microenvironment

## Abstract

Cancer-associated fibroblasts (CAFs) are key components in tumor microenvironment (TME). The secreted products of CAFs play important roles in regulating tumor cells and further impacting clinical prognosis. This study aims to reveal the relationship between CAF-secreted cytokines and breast cancer (BC) by constructing the risk signature. We performed three algorithms to reveal CAF-related cytokines in the TCGA BC dataset and identified five prognosis-related cytokines. Then we used single-cell RNA sequencing (ScRNA-Seq) datasets of BC to confirm the expression level of these five cytokines in CAFs. METABRIC and other independent datasets were utilized to validate the findings in further analyses. Based on the identified five-cytokine signature derived from CAFs, BC patients with high-risk score (RS) had shorter overall survival than low-RS cases. Further analysis suggested that the high-RS level correlated with cell proliferation and mast cell infiltration in BCs of the Basal-like subtype. The results also indicated that the level of RS could discriminate the high-risk BC cases harboring driver mutations (i.e., PI3KCA, CDH1, and TP53). Additionally, the status of five-cytokine signature was associated with the frequency and molecular timing of whole genome duplication (WGD) events. Intratumor heterogeneity (ITH) analysis among BC samples indicated that the high-RS level was associated with the increase of tumor subclones. This work demonstrated that the prognostic signature based on CAF-secreted cytokines was associated with clinical outcome, tumor progression, and genetic alteration. Our findings may provide insights to develop novel strategies for early intervention and prognostic prediction of BC.

## Introduction

Breast cancer (BC) is one of the most common cancers of women and remains a major cause of cancer-associated death worldwide (1). Despite the improvement of both early detection and therapies of BC, patients are still facing a severe challenge in terms of poor prognosis. Based on the high-throughput transcriptional data, analysis of molecular typing is often performed to indicate differential pathological features and clinical prognosis among BC patients, for which PAM50 subtyping was most widely used (2, 3). Although these molecular subtypes were derived from the mathematic clustering, the prognosis among BC cases within each subtype still vary widely. Therefore, it is of great clinical significance to explore novel prognostic signatures.

The tumor microenvironment (TME) has been recognized to play an important role in the initiation and progression of BC over the past decades (4). Cancer-associated fibroblasts (CAFs) are one of the most dominant components in the tumor stroma and have a tremendous influence on remodeling the extracellular matrix (ECM) structure (5). Previous studies have demonstrated the pro-tumorigenic role of CAFs in accelerating tumor proliferation, angiogenesis, and metastasis of many types of tumor, especially for BC (6, 7). However, the exact origin and biology of CAFs and the association between CAFs and clinical outcomes are not fully understood. CAF-mediated molecular mechanisms mainly rely on multilayered communications of CAFs with the surrounding cancer cells and other components within the TME (8). Therefore, utilizing CAF-secreted cytokines to predict therapeutic effect and clinical prognosis is worth further investigation.

Risk signatures were widely used to predict prognostic outcomes in cancer research. Van De Vijver et al. firstly conducted a 70-gene signature that is closely associated with survival of patients with BC (9). Furthermore, kinds of risk signatures were constructed among types of cancer (10, 11), which were proved to be more precise in predicting clinical prognosis than traditional methods, including pathological and imaging estimations (12). In addition to the gene expression data of tumor tissues, chemokines or cytokines were indicated to be a novel strategy for developing risk signatures, which got a weak dependence on gene numbers and showed great potential of non-invasive detection (13, 14). In the area of breast cancer research, the role of TME-related molecular regulation has not been fully revealed. We therefore considered CAF-related cytokines into the construction of risk signature to estimate a novel strategy for predicting the prognosis of patients with BC.

In this study, we identified five prognosis-related cytokines that were highly expressed in CAFs of BC. Through the Cox hazard model, we constructed a novel risk signature based on the expression level of the five cytokines in the TCGA BC dataset. Following analyses suggested that the five-cytokine signature was associated with clinical outcome, tumor cell proliferation program, immune cell activation, and genomic alteration, which were further validated in METABRIC and other independent datasets.

## Materials and Methods

### Study Design

This study included gene expression, somatic alteration, and clinical outcomes data from TCGA (*n* = 1,091) and METABRIC (*n* = 1,904) datasets, which were used for the training and validation of the five-cytokine signature, respectively. To reveal the expression level of cytokines in types of tumor microenvironment (TME)-infiltrating cells, we performed analysis on two single-cell RNA sequencing (ScRNA-Seq) datasets of BC, which contain 565 and 24,271 cells derived from 11 and 5 BC tissue samples, respectively (15, 16). To further validate the prognostic relevance of five-cytokine signature, we included bulk RNA sequencing (RNA-Seq) and clinical prognosis data of two independent BC datasets (GSE20685 and GSE86166) for further analysis (17, 18). We also obtained the gene expression, TP53 mutation, and prognosis data of another BC dataset (GSE40954) to identify the variance between TCGA and METABRIC datasets (19). Additionally, detailed characteristics of included public access BC datasets are shown in **Table S1**.

### Inference of CAFs and Other Infiltrating Cells in TME

To quantify the proportion of CAFs in BC samples, we used EPIC (20), xCell (21), and MCP-counter (22) algorithms on the gene expression data. The EPIC analysis was conducted with default parameters, which indicated the calculated proportion of CAFs among BC samples. The results of xCell analysis performed on TCGA datasets were achieved from the previous study (21), and the results suggested the enrichment score of CAF-related gene signature. We conducted MCP-counter analysis using six CAF markers, including CD29, FAP, SMA, FSP1, PDGFRβ, and CAV1, according to the previous report (23). To quantify the proportions of other infiltrating immune cells in TME, we employed the CIBERSORT algorithm using the LM22 gene signature (24), which allows for the discrimination of 22 human immune cell phenotypes.

### Analysis of Single-Cell RNA Sequencing Data and the Construction of Risk Signature

To validate the specificity of the indicated cytokines in CAFs, we performed analyses on two single-cell RNA sequencing (ScRNA-Seq) datasets of BC to detect the expression level of cytokines in TME. Batch effects within the ScRNA-Seq data of GSE75688 were firstly removed using ComBat command from the R package sva (25). The expression profiles of 565 isolated cells were further reduced to two-dimensional representations by t-distributed stochastic neighbor embedding (tSNE) method (Rtsne R package). The cell type annotation was conducted according to the previous report (16). The ScRNA-Seq data of the other dataset were acquired from supplementary files as the Seurat object in R (**Table S1**) (15). We conducted a similar procedure for annotating cell types, and the results were shown using two-dimensional representations by tSNE method (Seurat R package).

**Figure 1:**
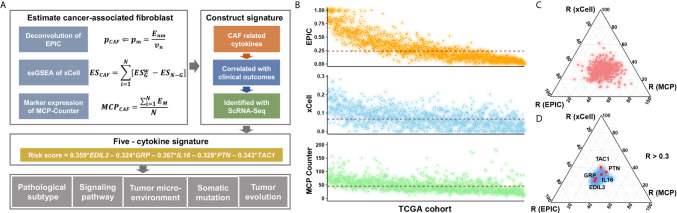
Study flow chart and the selection of candidate cytokines. **(A)** Where *E_nm_* is a matrix (n × m) of the n gene expression profiles from the m cell types; *V_n_* is the vector of all *n* genes expressed from the bulk sample to deconvolve; and *p* is a vector of the proportions from the *m* cell types in the given sample. *ES* ${}_{G}^{w}$ is the weighted enrichment score (ES) of a given signature *G*, and *ES_N-G_* is the ES of the remaining genes from the data set of *N* genes. *E_M_* is the gene expression of canonical CAF markers in BC datasets. **(B)** Inferred CAF proportion scores of TCGA BC cases by three algorithms (see *Methods*). EPIC, xCell, and MCP-Counter indicated the assumed CAF proportion, the enrichment score of CAF-related gene signature, and the relative expression level of CAF marker genes, respectively. And the red dotted line indicated median values. **(C)** The results of correlation analyses between all 695 cytokines and CAF proportion scores derived from algorithms. **(D)** The 106 CAF-related cytokines indicated medium and high correlation with CAF proportion scores (R > 0.3), and a total of five cytokines showed prognostic relevance.

The Cox proportional hazards model was utilized to construct the risk signature model. The BC cases of TCGA were grouped into high- or low-expression subgroup according to the median value of gene expression, and hazard ratios (HRs) and coefficients were then estimated of each candidate cytokines. Significant prognostic factors were further subjected to the construction of risk signature (**Figure 1A**).

### Construction of Cytokine-Immune Cell Network

The annotation of known cytokine and the cytokine-receptor network analyses were conducted using the CellTalker R package (26). The data of annotated 695 cytokines and corresponding 2,502 ligand-receptor pairs were retrieved from the TCGA dataset, and receptors of the five cytokines were included for the following analysis (**Table S2**). To calculate the association between the five-cytokine signature and TME, we analyzed the relationship between CIBERSORT-annotated immune cells and CellTalker-annotated receptors using Spearman rank correlation analysis, and the immune cell–related receptors were then considered into the construction of cytokine-immune cell network. Finally, the correlation between the cytokine and the certain type of immune cell was calculated using the following formula: $C=\Sigma_{i=1}^{n}R_{i}^{2}$, where *n* represents the number of cytokine-paired receptors and *R* represents the Spearman correlation coefficient between the receptor and the certain type of immune cell.

### Gene Set Variation Analysis on Gene Expression Data

Gene set variation analysis **(**GSVA) was utilized to obtain pathway scores based on RNA-seq data using the R package GSVA (27). A Wilcoxon rank sum test was performed subsequently to identify pathways differentially expressed among subgroups. *P* values were adjusted *via* the Benjamini-Hochberg procedure.

### Estimation of Genomic Features in the TCGA Cohort

We estimated the intratumor heterogeneity (ITH) using the mutant-allele tumor heterogeneity (MATH) method (28). The MATH score was calculated using the formula $MATH_{i}=\frac{MAD(AF_{i})}{Median(AF_{i})}\times 100$, where AF_i_ is a vector of the allele frequency (AF) of all mutations from sample *i*, and median absolute deviation (MAD) was denoted. Microsatellite instability (MSI) and DNA methylation data were achieved from the supplementary files of previous studies (29, 30). The whole genome duplication (WGD) events of BC genome in TCGA cohort were also achieved from the previous report (31).

### Statistical Methods

Statistical analyses were performed using R (v3.6.1). For comparisons of continuous variables between groups, Mann-Whitney U tests and Kruskal-Wallis H tests were used. For comparisons of categorical variables between groups, chi-squared or Fisher’s exact tests were utilized. *P* values were further adjusted for multiple hypothesis testing using the Benjamini-Hochberg method. To compare survival time and outcomes between groups, we used the log-rank test for Kaplan-Meier curves. All reported *P* values were two-sided. The differences were considered significant when the *P* value was <0.05 or the Benjamini-Hochberg false discovery rate (FDR) was <0.1.

## Results

### Five CAF-Related Cytokines Correlate With Clinical Prognosis

We applied three different algorithms to infer the CAF proportion scores in TCGA BC cases (**Figure 1B**), and the correlation analysis indicated consistent results among algorithms (**Figure S1A**). Candidate cytokines were filtered by correlation analyses using CAF proportion scores (**Figure S1B** and **Table S3**), and a total of 106 cytokines were indicated by all three algorithms (**Figure 1C** and **Figure S1C**). Among these CAF-related cytokines, we found five of them were associated with clinical outcomes (**Figure 1D**, **Figure S2A** and **Table S4**).

**Figure 2:**
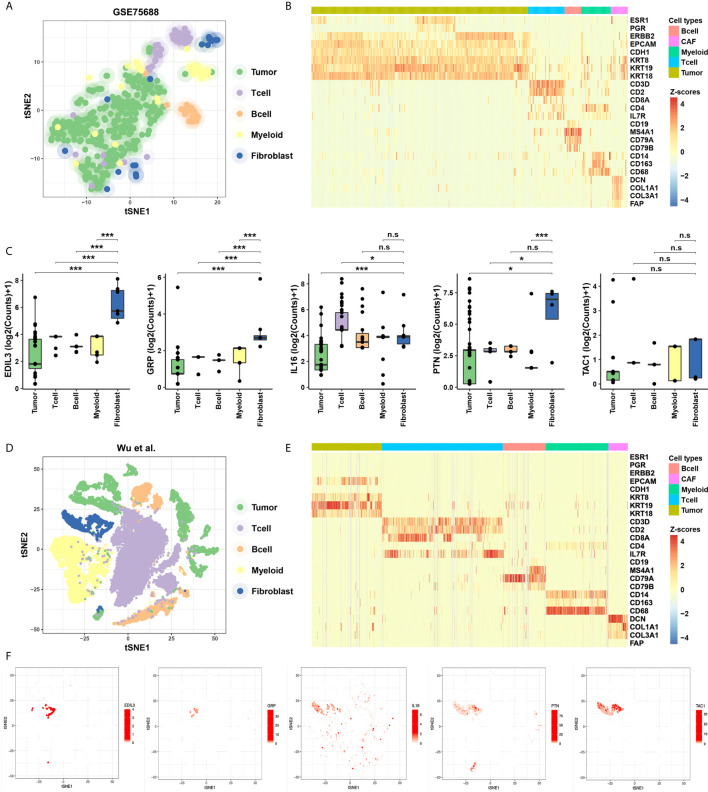
Identification of the specificity for the five cytokines in ScRNA-Seq data of BC. **(A)** Cellular populations identified in the GSE75688 dataset, and the tSNE projection of 565 cells showed the clusters with label names. Each dot corresponds to a single cell, colored according to cell type. **(B)** Heatmap of marker gene sets among tumor cells, T cells, B cells, Myeloid cells, and fibroblasts of GSE75688 dataset. **(C)** Comparisons on average expression of the five CAF-related cytokines. Bar, median; box, 25th to 75th percentiles (interquartile range, IQR); vertical line, data within 1.5 times the IQR. ***FDR < 0.001 and *FDR < 0.1. n.s., non-significant. **(D, E)** Clustering of 24,271 cells from the other ScRNA-Seq data of BC (see *Methods*) using tSNE method **(D)**, and the heatmap showed marker gene sets among different cell types **(E)**. **(F)** The five CAF-related cytokines were labeled in clusters by cell identity as represented in the tSNE plot.

To evaluate the specificity of expression levels of CAF-related cytokines, we analyzed the ScRNA-Seq data from GSE75688 and the other independent BC datasets (See *Methods*). Based on these two datasets, two-dimensional projection by tSNE grouped the cells distinctly into tumor cells, T cells, B cells, Myeloid cells, and CAFs (**Figures 2A, D**). We also explored the expression level of marker genes among cellular types, and fibroblast markers DCN, COL1A1, COL3A1, and FAP were highly expressed in CAFs as expected (**Figures 2B, E**). Further analyses suggested that EDIL3, GRP, PTN, and TAC1 were specifically expressed in CAFs, and IL16 displayed a salt-and-pepper expression pattern in both immune cells and CAFs (**Figures 2C, F**).

### Relevance Between Five-Cytokine Signature Status and Patient Outcomes

**Figure 3:**
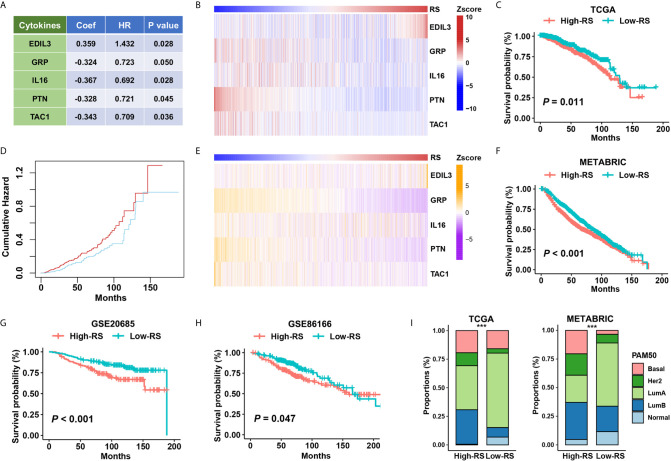
The association of five-cytokine signature status with clinical outcomes. **(A)** The features of the five-cytokine signature in the training data (TCGA). Coef, coefficient; HR, hazard ratio. **(B)** The heatmap plot for the expression level of each cytokine across TCGA samples. **(C, D)** The training results in the TCGA dataset. Kaplan‐Meier survival analysis and cumulative hazard curves using median cutoff RS. *P* value indicated by log-rank test. **(E, F)** The validation results in the METABRIC dataset. The heatmap plot for the expression level of included five cytokines across METABRIC samples, and Kaplan‐Meier survival analysis using median cutoff RS of five-cytokine signature. **(G, H)** Further validation in GSE20685 and GSE86166 datasets using median cutoff RS both suggested that the high-RS level was associated with the poor prognosis. **(I)** Grouping BC patients into five PAM50 subtypes (Lum A, Lum B, HER2-enriched, Basal-like, and Normal-like) based on five-cytokine signature status. ***P < 0.001.

We constructed a five-cytokine signature to indicate risk score (RS) using gene expression data of TCGA as the training dataset (**Figure 3A**; See *Methods*), and the analysis also indicated the robustness of the risk signature (**Figure S2B**). We observed the increasing trend of EDIL3 and decreasing trend of GRP, IL16, PTN, and TAC1 in this training dataset (**Figure 3B**), and the BC cases were further categorized into “High RS” (with high five-cytokine signature; higher median) or “Low RS” (with low five-cytokine signature; lower median) within the TCGA cohort. The analysis showed that high-RS cases had shorter overall survival and higher cumulative hazard than low-RS cases (**Figures 3C, D**). To validate these findings, we analyzed the gene expression and prognosis data of the METABRIC cohort, which were consistent with the training results in the TCGA cohort (**Figures 3E, F**). Furthermore, another two independent datasets, GSE20685 and GSE86166, were included to further validate the constructed five-cytokine signature, and the results suggested that high-RS cases had worse prognosis than low-RS ones (**Figures 3G, H**).

To address the relationship between the status of five-cytokine signature and commonly used PAM50 molecular subtypes, we calculated the proportions of PAM50 subtypes in high- and low-RS subgroups in both TCGA and METABRIC cohorts. The trend of more Basal-like, HER2-enriched, and Luminal B (LumB) breast tumors was found in high-RS cases, and more Luminal A (LumA) and Normal-like breast tumors were suggested in low-RS cases (**Figure 3I**).

### High RS Correlates With Cell Proliferation and Mast Cell Infiltration in Basal-Like Subtype

**Figure 4:**
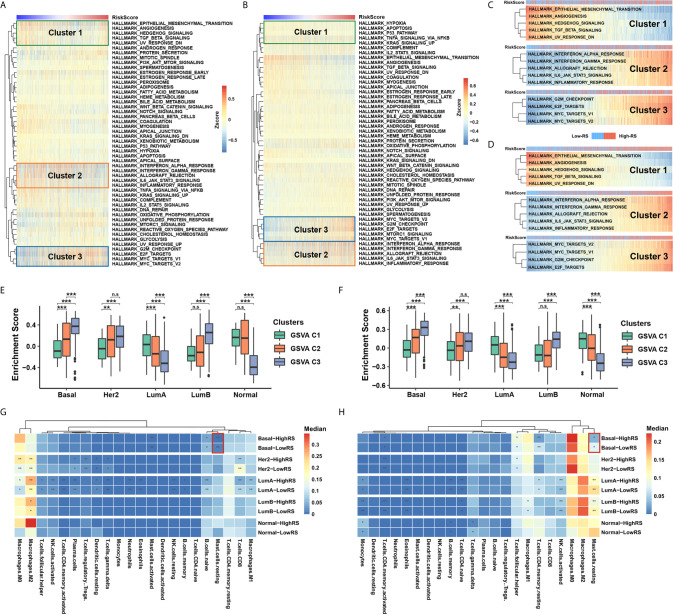
Five-cytokine signature contributes to molecular features of the Basal-like subtype. **(A, B)** GSVA analysis with hallmark gene sets indicated three pathway clusters in both TCGA **(A)** and METABRIC **(B)** cohorts (see *Methods*), which were epithelial–mesenchymal transition pathways (Cluster1), inflammation or stress response pathways (Cluster2), and cell proliferation pathways (Cluster3). **(C, D)** The RS level positively correlated Cluster1 and negatively associated Cluster2 and Cluster3 in both TCGA **(C)** and METABRIC **(D)** datasets. **(E, F)** Average enrichment score of the three distinct clusters among PAM50 subtypes in TCGA **(E)** and METABRIC **(F)** datasets. **(G, H)** Differential immune cell proportions of high- and low-RS groups within each PAM50 subtype in TCGA **(G)** and METABRIC **(H)** cohorts, respectively. *P* value indicated by Wilcoxon rank sum test and adjusted by Benjamini-Hochberg method. ***FDR < 0.001; **FDR < 0.01; *FDR < 0.1. n.s., non-significant.

To identify the underlying biological characteristics of the five-cytokine signature, we analyzed the association between the RS level and the activation of signaling pathways. The results of GSVA indicated that high RS correlated with the activation of epithelial–mesenchymal transition pathways (Cluster1), inflammation or stress response pathways (Cluster2), and cell proliferation pathways (Cluster3) in both TCGA and METABRIC cohorts, which are shown in **Figures 4A, B**, respectively. The RS level of the five-cytokine signature indicated a positive relationship with Cluster1 and negative associations with Cluster2 and Cluster3 in both TCGA and METABRIC datasets (**Figures 4C, D**). For BC cases among different PAM50 subtypes, the identified clusters demonstrated distinct enrichment patterns, in which Cluster3 was found to be significantly enriched in Basal-like subtype (**Figures 4E, F**).

Utilizing the network analysis between ligands and receptors (see *Methods*), we demonstrated the strong correlation between the five-cytokine signature and other cell types in TME (**Figures S3A, B**). In terms of different types of tumor-infiltrating immune cells, we found that resting mast cells were decreased in high-RS BC samples of Basal-like subtype, which was also validated in METABRIC cohort (**Figures 4G, H** and **Figure S3C**).

### Five-Cytokine Signature Discriminates High-Risk Patients Harboring PIK3CA, CDH1, and TP53 Mutations

**Figure 5:**
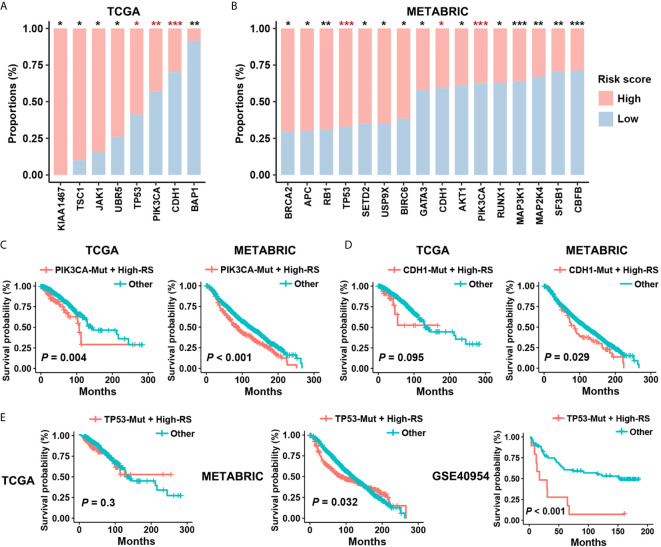
Prognostic risk of high-RS patients harboring driver mutations. **(A, B)** Non-synonymous mutations of driver genes (Cancer Gene Census, v84) within high- or low-RS groups in TCGA and METABRIC cohorts. The consistent trends were observed among TP53, PIK3CA, and CDH1 mutations. *P* value indicated by Fisher’s exact test. ***P < 0.001; **P < 0.01; *P < 0.05. **(C)** High-RS patients harboring PIK3CA mutations had significant poor prognosis in both TCGA and METABRIC cohort. **(D, E)** High-RS level contributed more prognostic risk to CDH1 or TP53 mutated cases in the METABRIC dataset, and GSE40954 validated the higher risk of high-RS cases harboring TP53 mutations. *P* value suggested by log-rank test.

Concurrent driver gene mutations had an important impact on BC prognosis (32). Our results suggested that certain driver mutations correlated with the status of five-cytokine signature. We found that PIK3CA and CDH1 mutations enriched in the low-RS group, but TP53 mutations were associated with a high proportion of high-RS cases in both cohorts (**Figures 5A, B**). Additionally, these driver mutations demonstrated distinct relationships with pathway clusters (**Figures S3D, E**). The analyses indicated that TP53 mutations correlated with the enrichment of Cluster2 and Cluster3, and PI3KCA mutations contributed to the activation of Cluster1.

Although PIK3CA and CDH1 mutations are the therapy target and the risk factor in BC separately (33, 34), survival analysis suggested weak relevance between PIK3CA or CDH1 mutations and the clinical outcomes in both TCGA and METABRIC datasets (**Figures S4A, B**). However, after considering the status of five-cytokine signature, high-RS cases harboring PIK3CA mutations showed significant worse prognosis (**Figure 5C**), and CDH1 mutated cases with high-RS level indicated poor clinical outcomes (**Figure 5D**). Notably, although TP53 mutations were also found to be enriched in the high-RS cases (**Figures 5A, B**), the heterogenous prognostic relevance of TP53 mutations among different datasets was observed (**Figure S4C**). Further survival analyses suggested that the use of five-cytokine signature status improved the prediction of clinical outcomes in TP53-mutated BC patients (**Figure 5E**). All these results indicated the potential molecular crosstalk between tumor somatic mutations and CAFs, which could further impact the clinical prognosis.

### The Impact of the Five-Cytokine Signature on Tumor Evolution in BC

**Figure 6:**
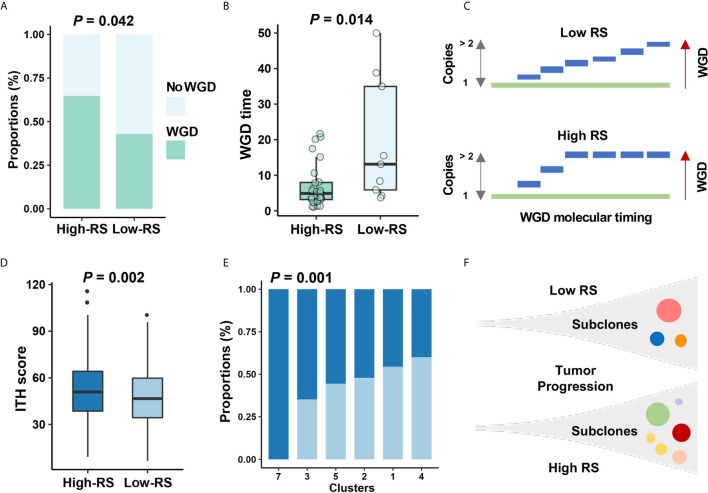
The relationship between five-cytokine signature and tumor evolution history. **(A, B)** In TCGA data, more WGD events (see *Methods*) were found to be enriched in high-RS BC samples, and the shorter WGD timing (see *Methods*) was further observed in the high-RS group, which were suggested by Fisher’s exact test and Wilcoxon rank sum test, respectively. **(C)** Schematic diagram indicated that high-RS correlated with more proportions of WGD events and earlier WGD timing before the appearance of the MRCA among all BC cells. **(D, E)** The higher ITH scores and more mutational clusters were revealed in the high-RS group using MATH algorithm (see *Methods*). *P* values were indicated by Wilcoxon rank sum test and Fisher’s exact test separately. **(F)** Schematic diagram indicated that high-RS tumors harbored more subclones during the tumor progression of BC.

To investigate the impact of high-RS level on tumor evolution, we estimated whole genome duplication (WGD) and intratumor heterogeneity (ITH) within high- or low-RS tumor samples. Whole genome duplication (WGD) is the result of estimating the ratio of duplicated to non-duplicated mutations when the genome gain happened during clonal evolution (31), which indicated the molecular timing before the appearance of the most recent common ancestor (MRCA). Our results suggested that high-RS level was associated with high frequency and earlier timing of WGD events (**Figures 6A–C**), and we also found that WGD events correlated with poor prognosis, which was consistent with the previous study (**Figure S5A**) (35). To validate these findings, we performed analyses on ovary adenocarcinoma (OV) data in TCGA, which indicated the relevance between the high-RS level and earlier timing of WGD events (**Figures S5B, C**).

Previous reports for early-stage BC demonstrated the high homogeneity in clonal expansion (36), low ITH (37), and punctuated evolution (38). Accordingly, we analyzed the clonal expansion of BC under the impact of high-RS level. The ITH analysis suggested the association between five-cytokine signature status and ITH scores in BC (**Figure 6D**), and the high-RS level was found to be related with the increase of tumor subclones (**Figures 6E, F**). Microsatellite instability (MSI) and DNA methylation analyses further validated that the high-RS level was related with more genomic and epigenomic alterations (**Figures S5D, E**).

## Discussion

In this study, we examined the effects of five-cytokine signature derived from CAFs on clinical prognosis of BC patients, and we tested the hypothesis that five-cytokine signature correlated with molecular phenotype, TME, somatic mutation, and tumor evolution. This risk signature model was constructed using TCGA datasets and validated in METABRIC and other independent datasets, which suggested the robustness of this five-cytokine signature. All these results demonstrated that CAF-released cytokines play an important role in tumor progression of BC. Better understanding of this intracrine environment contributes to reveal the contradictions surrounding the effects among CAFs, immune cells, and tumor cells in BC.

CAFs were found to secrete numerous chemokines or cytokines, including TGF-β, IL-6, IL-8, IL-13, CXCL12, CXCL14, and VEGF (8). In addition to these known cytokines, our results demonstrated that EDIL3, GRP, IL16, PTN, and TAC1 were specifically or highly expressed within CAFs and could act as prognostic factors of BC. For these indicated CAF-related cytokines, the high expression level of EDIL3 correlated with poor prognosis in multiple tumors (39, 40); GRP was revealed to participate in CAF subtype transition in pancreatic ductal adenocarcinoma (PDAC) (41); IL16 polymorphisms were suggested to be associated with the high risk in types of cancer (42, 43), which were found to be regulated by the expression quantitative trait loci (eQTL) according to a genome-wide association study (GWAS) (44); PTN was also indicated to act as the downstream target of CDKN1A for a critical role in BC chemoresistance. In addition, our results demonstrated the relevance between the five-cytokines signature and TME. Previous studies indicated that CAF-secreted cytokines suppress the recruitment of immune cells, including abolishing CTLA-4 (45), reducing PD-L1 (46), and inducing Tregs in the tumor stroma to create a tumor-promoting microenvironment (47). Therefore, the immune features of high-RS cases were warranted for further research. In summary, few studies demonstrated the molecular mechanism and the immune correlation of the five cytokines within our risk signature. Although the underlying mechanisms were not able to be revealed in this study, our present results suggested that these CAF-secreted cytokines might be worth exploring further.

Notably, the heterogeneities of CAFs existed in BC, which might impact the sensitivity of the CAF-related prognostic signature. Pietras et al. classified breast CAFs into three different subtypes, which were named vCAFs, mCAFs, and dCAFs, based on ScRNA-Seq of CAFs isolated from the mice model (48). All these CAF subtypes correlated to distinctive functional programs and acted as independent prognostic factors of BC (48). Furthermore, Costa et al. identified four subsets of CAFs in BC, which were referred to as CAF-S1, CAF-S2, CAF-S3, and CAF-S4 (23). CAF-S1 might promote an immunosuppressive environment in the triple negative BC (TNBC) and was characterized with a poor prognosis (23). A recent study indicated two main CAF subpopulations in breast tumors, where their ratio is associated with disease outcome and is particularly correlated with genomic variations in TNBC (49). In addition to driver mutations, the expression level of TP53 was also found to be correlated with the activation of specific CAF subtypes (50). In summary, these findings suggested complex interactions among CAF subtypes, tumor cell mutation, and the expression of driver genes. The use of the computational methods in bulking sequencing data might not work effectively in the discrimination of heterogenous CAFs, but the widely used single-cell sequencing would improve the specificity and robustness of the cytokines-based signature in future studies.

In conclusion, our results demonstrated that the five-cytokine signature was associated with clinical outcomes, tumor cell proliferation program, immune cell activation, and genomic alterations in BC. Our data suggested that the RS level derived from the five-cytokine signature could serve as a predictive indicator for BC prognosis, and these findings might provide insights to develop novel treatment strategies for BC.

## Data Availability Statement

Publicly available datasets were analyzed in this study. These data can be found here: https://www.cbioportal.org/study/summary?id=brca_metabric%2Cbrca_tcga_pan_can_atlas_2018, https://www.ncbi.nlm.nih.gov/geo/query/acc.cgi?acc=GSE75688, https://www.ncbi.nlm.nih.gov/geo/query/acc.cgi?acc=GSE20685, https://www.ncbi.nlm.nih.gov/geo/query/acc.cgi?acc=GSE86166, https://www.ncbi.nlm.nih.gov/geo/query/acc.cgi?acc=GSE40954, and https://www.embopress.org/doi/abs/10.15252/embj.2019104063

## Author Contributions

CS: study conceive and design, data analysis and interpretation, and drafting of the manuscript. SW: study design, data analysis, and drafting of the manuscript. YZ, FY, TZ, and FM: data analysis and curation. MY, YYa, YH, ZF, JL, XH, and HW: study supervision, review and editing. YYi and WL: study design and revision of the manuscript, project administration, and funding acquisition. All authors contributed to the article and approved the submitted version.

## Funding

This work was supported by the National Science Foundation of China (81972484, 81772475, 81972484, 81902704), Youth medical talent project in science and education of Jiangsu Province (QNRC2016855), Collaborative innovation center for tumor individualization focuses on open topics of Nanjing Medical University (JX21817902/008), High-level innovation team of Nanjing Medical University (JX102GSP201727), Project of China Key Research and Development Program Precision Medicine Research (2016YFC0905901), and Key Medical Talents of Jiangsu Province (ZDRCA2016023).

## Conflict of Interest

The authors declare that the research was conducted in the absence of any commercial or financial relationships that could be construed as a potential conflict of interest.

## Publisher’s Note

All claims expressed in this article are solely those of the authors and do not necessarily represent those of their affiliated organizations, or those of the publisher, the editors and the reviewers. Any product that may be evaluated in this article, or claim that may be made by its manufacturer, is not guaranteed or endorsed by the publisher.

## Supplementary Material

Supplementary Figure 1 Inferring CAF proportion scores in TCGA BC data using three algorithms and screening CAF-related cytokines. **(A)** The consistency among the CAF proportion scores of three algorithms, which were indicated by Spearman rank correlation analysis. **(B, C)** The CAF-related cytokines were estimated according to the three algorithms.

Supplementary Figure 2 Prognostic features of five cytokines in the risk signature. **(A)** Kaplan‐Meier survival analysis of the five cytokines that were included in the risk signature using median cutoff expression level in the TCGA BC dataset. **(B)** A drop-out model was used to test the robustness of the prognostic relevance. The exclusion of EDIL3, IL16, PTN, and TAC1 could weaken the ability of predicting the prognosis, but the exclusion of GRP might lead to overfitting. *P* values indicated by log-rank test.

Supplementary Figure 3 The association of five-cytokine signature status with immune cells in TME. **(A, B)** Constructed risk signature–immune cell network in TCGA **(A)** and METABRIC **(B)** cohorts, respectively. **(C)** The differential expression level of resting mast cells in high- and low-RS groups within two datasets. **(D, E)** The relevance between GSVA pathway clusters and driver mutations in TCGA and METABRIC datasets. *P* value, Wilcoxon rank sum test. ****P* < 0.001 and ***P* < 0.01. n.s., non-significant.

Supplementary Figure 4 Prognostic relevance of PIK3CA, CDH1, and TP53 mutations in BC. **(A, B)** Kaplan‐Meier survival analyses on PI3KCA and CDH1 mutated patients in TCGA and METABRIC datasets. **(C)** Survival analyses on BC cases harboring TP53 mutations in three datasets. *P* value suggested by log-rank test.

Supplementary Figure 5 The relationships between five-cytokine signature status and genomic and epigenomic alterations. **(A)** The prognostic relevance of WGD events in the TCGA BC dataset. **(B)** The shorter WGD timing (see *Methods*) was revealed among high-RS cases in the TCGA OV dataset. **(C)** Survival analysis indicated poor clinical outcomes of OV cases with WGD events. **(D)** The comparison of microsatellite instability (MSI) and microsatellite stable (MSS) based on five-cytokine signature status in TCGA cohort. **(E)** The comparison of DNA methylation status based on the five-cytokine signature in METABRIC cohort. ****P* < 0.001.

Supplementary Table 1 Characteristics and detailed information of included datasets.

Supplementary Table 2 The ligand–receptor pairs of the five cytokines according to the annotation of CellTalker R package.

Supplementary Table 3 The results of correlation analysis between cytokines and inferred CAF proportions among three algorithms (P < 0.05).

Supplementary Table 4 The results of Cox proportional hazards model among 106 candidate cytokines.
