# Supplementary figures and images for: Risk Signature of Cancer-Associated Fibroblast–Secreted Cytokines Associates With Clinical Outcomes of Breast Cancer
Source: Front Oncol. 2021 Jul 28;11:628677. doi: 10.3389/fonc.2021.628677 (PMC8356635; doi:10.3389/fonc.2021.628677)

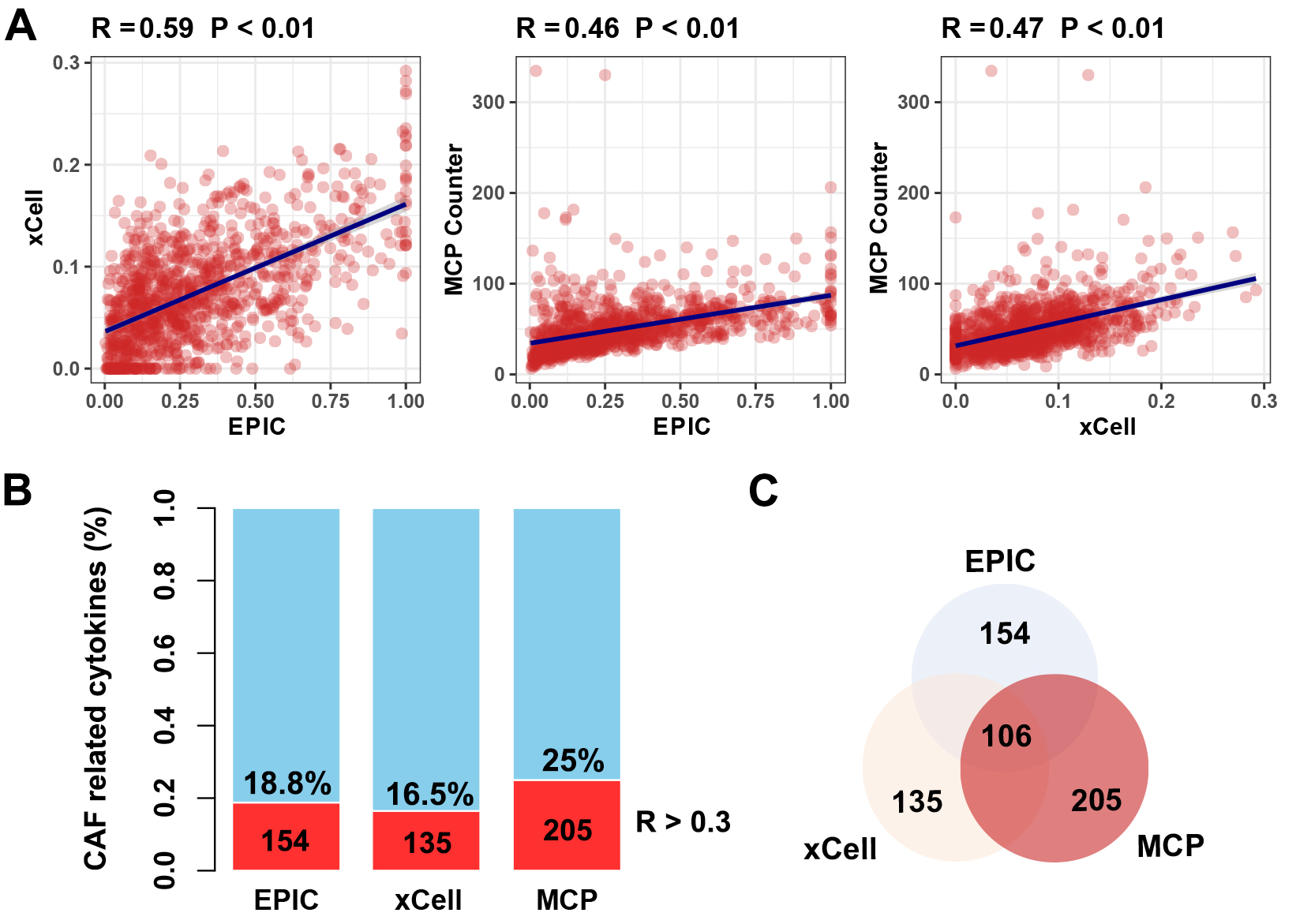

Supplement: Supplementary Figure 1 — Inferring CAF proportion scores in TCGA BC data using three algorithms and screening CAF-related cytokines. (A) The consistency among the CAF proportion scores of three algorithms, which were indicated by Spearman rank correlation analysis. (B, C) The CAF-related cytokines were estimated according to the three algorithms. [file Image_1.jpeg]

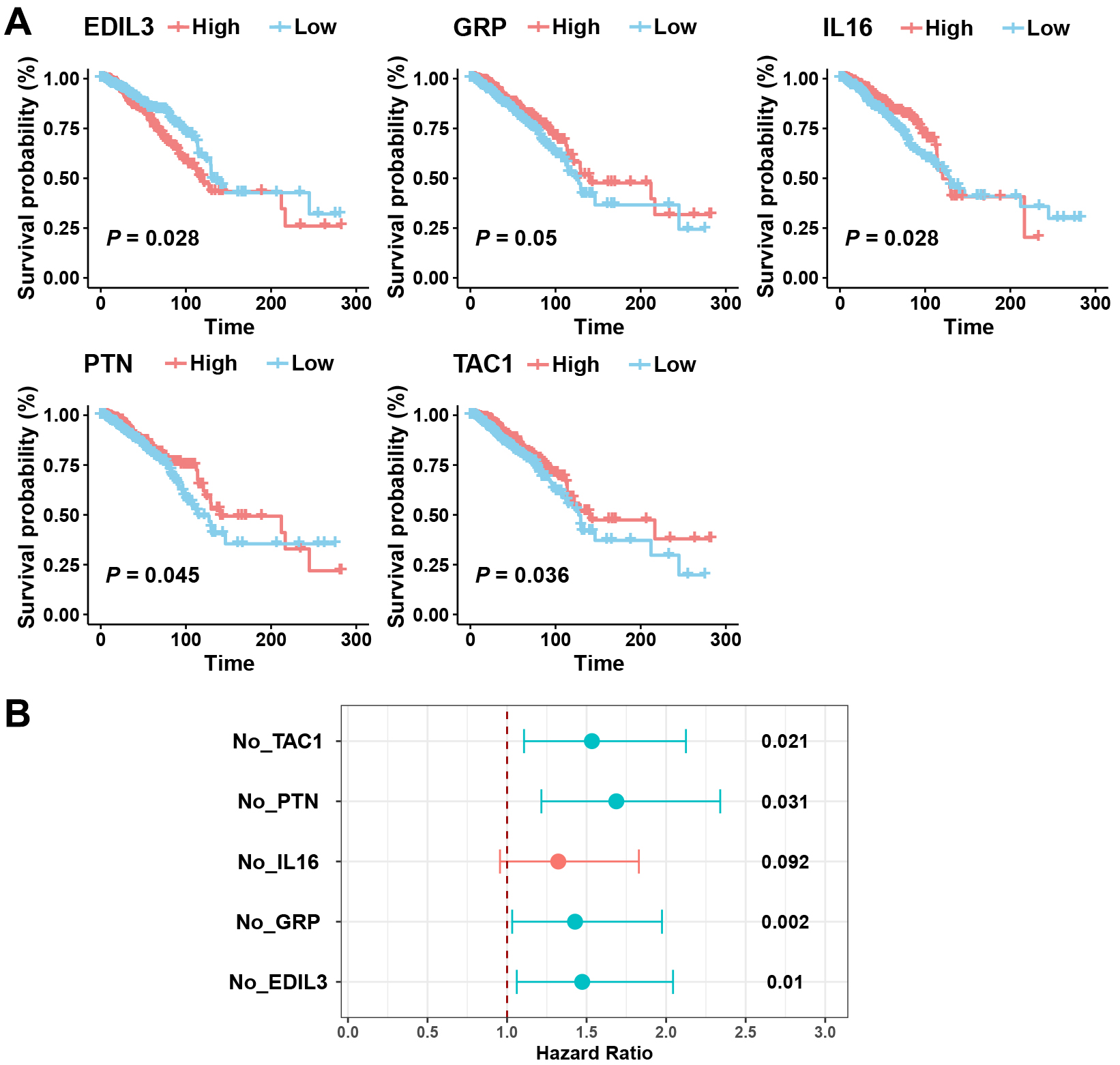

Supplement: Supplementary Figure 2 — Prognostic features of five cytokines in the risk signature. (A) Kaplan‐Meier survival analysis of the five cytokines that were included in the risk signature using median cutoff expression level in the TCGA BC dataset. (B) A drop-out model was used to test the robustness of the prognostic relevance. The exclusion of EDIL3, IL16, PTN, and TAC1 could weaken the ability of predicting the prognosis, but the exclusion of GRP might lead to overfitting. P values indicated by log-rank test. [file Image_2.jpeg]

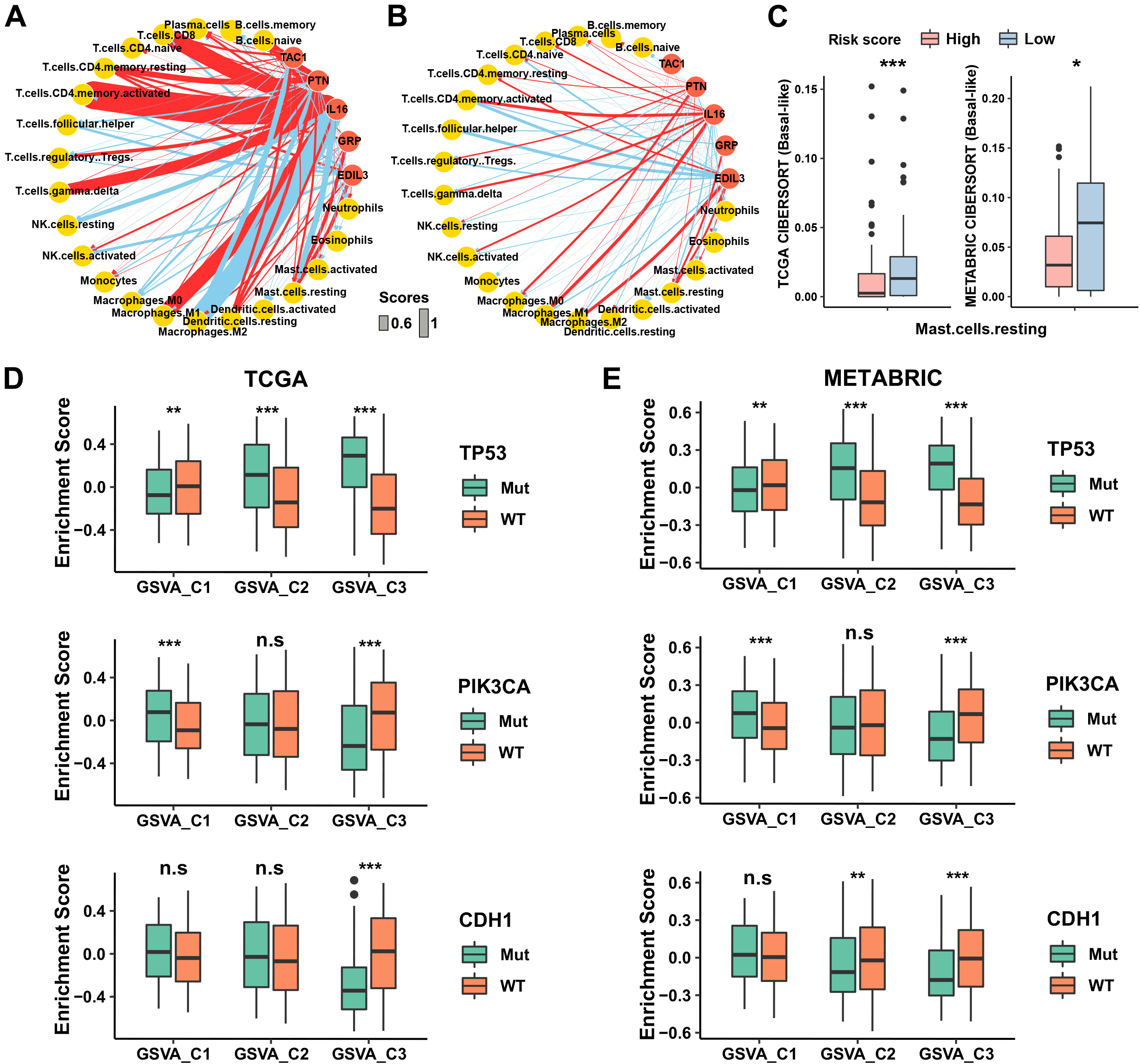

Supplement: Supplementary Figure 3 — The association of five-cytokine signature status with immune cells in TME. (A, B) Constructed risk signature–immune cell network in TCGA (A) and METABRIC (B) cohorts, respectively. (C) The differential expression level of resting mast cells in high- and low-RS groups within two datasets. (D, E) The relevance between GSVA pathway clusters and driver mutations in TCGA and METABRIC datasets. P value, Wilcoxon rank sum test. ***P < 0.001 and **P < 0.01. n.s., non-significant. [file Image_3.jpeg]

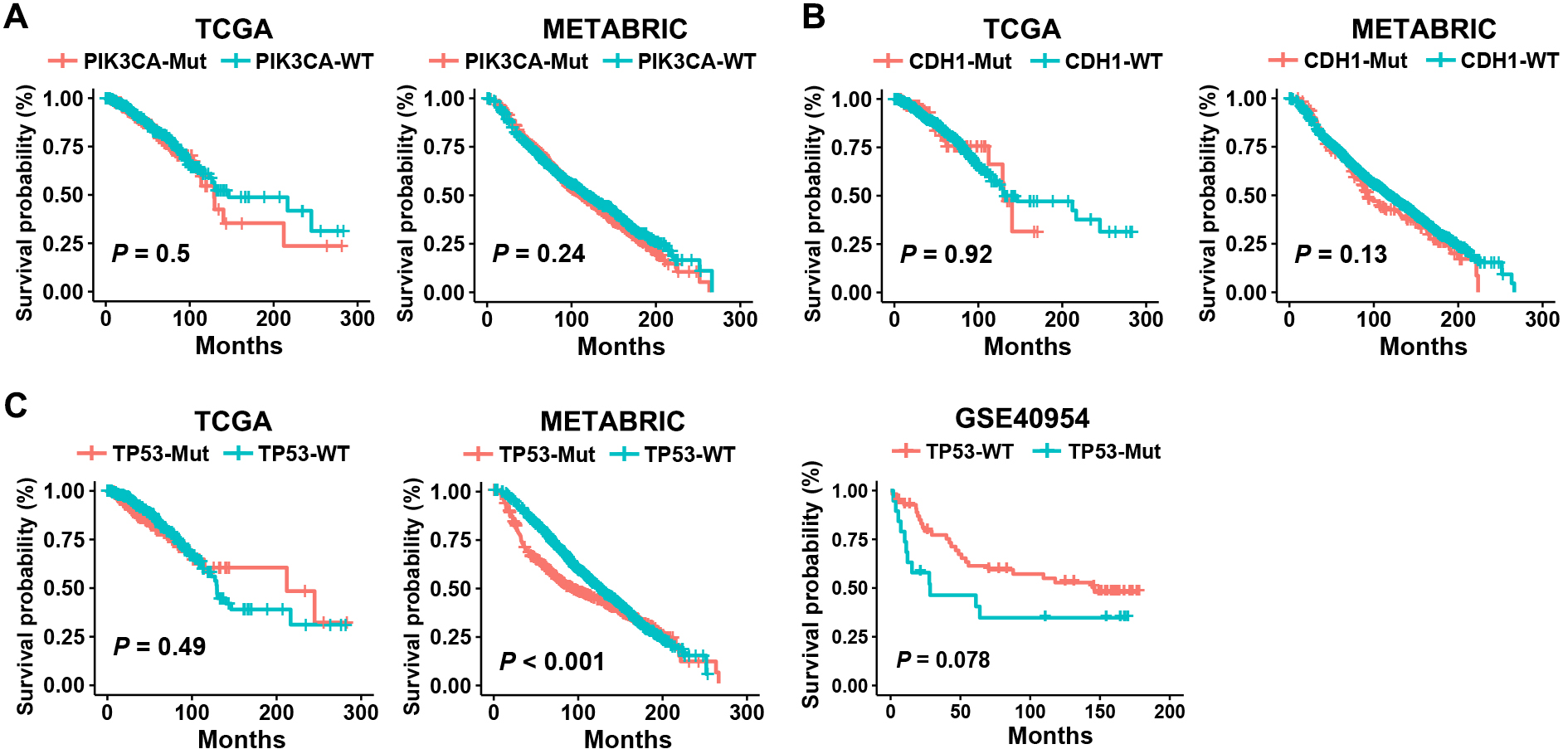

Supplement: Supplementary Figure 4 — Prognostic relevance of PIK3CA, CDH1, and TP53 mutations in BC. (A, B) Kaplan‐Meier survival analyses on PI3KCA and CDH1 mutated patients in TCGA and METABRIC datasets. (C) Survival analyses on BC cases harboring TP53 mutations in three datasets. P value suggested by log-rank test. [file Image_4.jpeg]

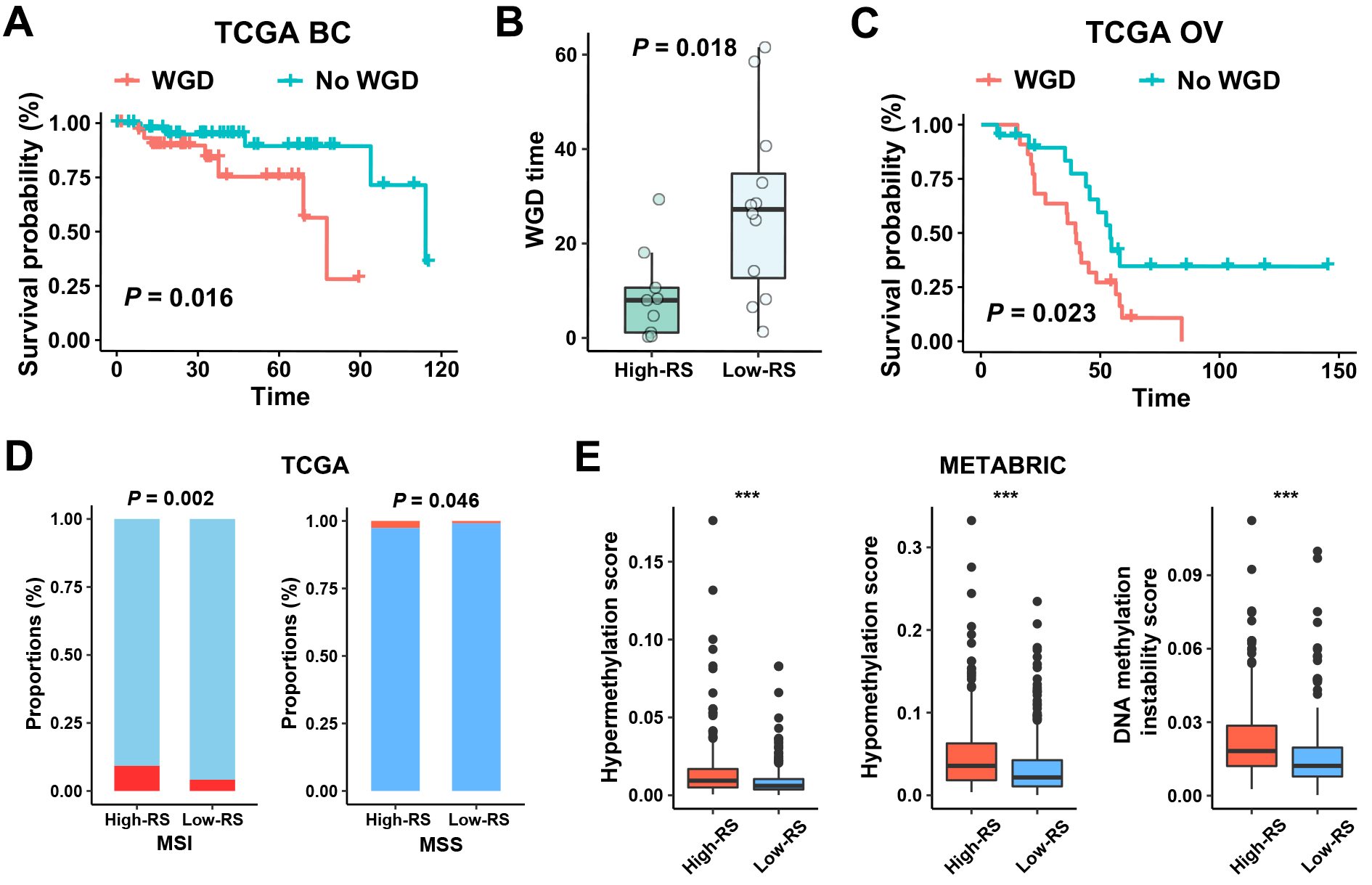

Supplement: Supplementary Figure 5 — The relationships between five-cytokine signature status and genomic and epigenomic alterations. (A) The prognostic relevance of WGD events in the TCGA BC dataset. (B) The shorter WGD timing (see Methods) was revealed among high-RS cases in the TCGA OV dataset. (C) Survival analysis indicated poor clinical outcomes of OV cases with WGD events. (D) The comparison of microsatellite instability (MSI) and microsatellite stable (MSS) based on five-cytokine signature status in TCGA cohort. (E) The comparison of DNA methylation status based on the five-cytokine signature in METABRIC cohort. ***P < 0.001. [file Image_5.jpeg]
